# Metabolism, ATP production and biofilm generation by *Staphylococcus epidermidis* in either respiratory or fermentative conditions

**DOI:** 10.1186/s13568-020-00966-z

**Published:** 2020-02-11

**Authors:** Ulrik Pedroza-Dávila, Cristina Uribe-Alvarez, Lilia Morales-García, Emilio Espinoza-Simón, Ofelia Méndez-Romero, Adriana Muhlia-Almazán, Natalia Chiquete-Félix, Salvador Uribe-Carvajal

**Affiliations:** 1 Department of Genetics and Molecular Biology, Instituto de Fisiología Celular, Universidad Nacional Autónoma de México (UNAM), Mexico City, Mexico; 2 Centro de Investigación y Desarrollo en Alimentos (CIAD), Hermosillo, Sonora Mexico

**Keywords:** *Staphylococcus epidermidis*, Oxygen concentration, Metabolism, Biofilms, Rate of oxygen consumption, Fermentation

## Abstract

*Staphylococcus epidermidis* is a Gram-positive saprophytic bacterium found in the microaerobic/anaerobic layers of the skin that becomes a health hazard when it is carried across the skin through punctures or wounds. Pathogenicity is enhanced by the ability of *S. epidermidis* to associate into biofilms, where it avoids attacks by the host and antibiotics. To test the effect of oxygen on metabolism and biofilm generation, cells were cultured at different oxygen concentrations ([O_2_]). As [O_2_] decreased, *S. epidermidis* metabolism went from respiratory to fermentative. Remarkably, the rate of growth decreased at low [O_2_] while a high concentration of ATP ([ATP]) was kept. Under hypoxic conditions bacteria associated into biofilms. Aerobic activity sensitized the cell to hydrogen peroxide-mediated damage. In the presence of metabolic inhibitors, biofilm formation decreased. It is suggested that at low [O_2_] *S. epidermidis* limits its growth and develops the ability to form biofilms.

## Introduction

Saprophytic microorganisms control pathogenic bacteria, digest nutrients and synthesize coenzymes, prosthetic groups and amino acids (Foster et al. 2005; Berg 1996; Sender et al. 2016). In the skin, *Staphylococcus epidermidis* inhibits colonization by *Staphylococcus aureus* or *Streptococcus pyogenes* secreting antimicrobial compounds and proteases (Cogen et al. 2010; Iwase et al. 2010). In the skin, *S. epidermidis* inhabits the epidermis, dermis and the nearly anoxic sebaceous glands (Grice and Segre 2011).

*Staphylococcus epidermidis* is frequently introduced through wounds and surgical procedures. A recent study reported the presence of antibiotic-resistant *S. epidermidis* strains in 46% of hospital secondary infections (Chabi and Momtaz 2019). Many of these strains were resistant to at least three antibiotics (Chabi and Momtaz 2019). Indeed, many antibiotics have to be tested in order to treat *S. epidermidis* nosocomial infections (Roujansky et al. 2020). *S. epidermidis* is also found frequently in implanted devices such as valves and catheters. There is an active search for materials to coat implant surfaces which may prevent biofilm formation (Rabin et al. 2015). Among these, zirconium nitride has shown promise in orthopaedic implants (Pilz et al. 2019), while sphingosine coating is being used with success on implant titanium-surfaces (Beck et al. 2019). Inside the body, this bacterium has to face attack from the immune system, high [O_2_] (Fang et al. 2016) and antibiotics (Leid 2009), most likely triggering a stress response. Within the organism, *S. epidermidis* may find areas with low [O_2_], similar to its natural habitat; it is likely that the bacterium will make an effort to remain in the hypoxic area, adhering to the surface and organizing into biofilms (Lewis 2007; Uribe-Alvarez et al. 2016). In regard to hypoxic environments within the host, these are often found at or near artificial devices such as catheters or prosthetic valves, where biofilms may force removal of implanted devices (Fey and Olson 2010; Büttner et al. 2015).

Understanding the *S. epidermidis* response to different [O_2_] would help optimize treatments (Cotter et al. 2009). We have reported that growing *S. epidermidis* at different [O_2_] modifies expression of respiratory chain enzymes and the ability to form biofilms (Uribe-Alvarez et al. 2016). At high [O_2_], cytochrome oxidases and NADH dehydrogenases are abundant and biofilms are minimal. In contrast, [O_2_] depletion increases nitrate reductase expression and association into biofilms (Uribe-Alvarez et al. 2016).

Here, the effect of [O_2_] on both, the aerobic and anaerobic metabolism of *S. epidermidis* was evaluated, together with [ATP]. In addition, the sensitivity of *S. epidermidis* to the toxic effects of hydrogen peroxide was tested. In each case, the biofilm-forming activity of cells was measured (Lewis 2007). When ATP synthesis was inhibited to different degrees by inhibitors of respiration (cyanide) (Uribe-Alvarez et al. 2016) or glycolysis (1,4-bisphosphobutane) (Hartman and Barker 1965; Rosas-Lemus  et al. 2016a), biofilm formation also decreased. It is suggested that *S. epidermidis* associates into biofilms as a strategy to avoid high [O_2_].

## Materials and methods

### Bacterial strain and growth media

*Staphylococcus epidermidis* strain ATCC 12228 was a kind donation from Dr. Juan Carlos Cancino Díaz (Instituto Politécnico Nacional, México). A loophole from the bacterium was suspended in 5 mL of 3% tryptic soy broth (Fluka, Sigma) and incubated at 37 °C for 24 h. Pre-cultures were added to 1 L LB medium (1% tryptone, 0.5% yeast extract, 1% NaCl) plus 2% glucose and incubated 24 h at 30 °C under aerobic (shaking 150 rpm), microaerobic (5% CO_2_, no agitation) or anaerobic (static in oxygen-depleted sealed acrylic chamber) conditions. Then the cells were washed three times at 5000×*g* for 10 min with distilled water and resuspended in 10 mM HEPES pH 7.4 (Uribe-Alvarez et al. 2016).

### Cytoplasmic extracts

All procedures were conducted at 4 °C. Cells (grown under aerobic, microaerobic or anaerobic conditions) were centrifuged at 5000×*g* for 10 min, washed three times with distilled water and resuspended in 50 mL 10 mM HEPES, pH 7.4, supplemented with one tablet of protease-inhibitor cocktail (Complete) and 1 mM PMSF. Cells were disrupted by sonication using a Sonics VibraCell sonicator (Sonics & materials, Inc., Newtown, CT) 7 × 20 s with 20 s intervals. To remove unbroken cells the suspension was centrifuged at 10,000×*g* for 10 min and the supernatant was recovered.

### Protein concentration

Protein concentrations from intact *S. epidermidis* cells were determined by the biuret method (Gornall et al. 1949). Absorbance (540 nm) was measured in a Beckman-Coulter DU50 spectrophotometer. For cytoplasmic extracts, protein concentration was measured by Bradford at 595 nm, using 1 or 2 µL aliquots of the sample in a PolarStar Omega (BMG labtech, Ortenberg, Germany) (Bradford 1976).

### Rate of oxygen consumption

The rate of oxygen consumption was measured in 10 mM HEPES pH 7.4 plus the indicated respiratory substrate. Bacteria, 0.5 mg prot mL^−1^ were added to a water-jacketed 1 mL chamber at 37 °C equipped with a Clark type electrode connected to a Strathkelvin model 782 oxymeter. Data were analyzed using the 782 Oxygen System Software (Warner/Strathkelvin Instruments) (Uribe-Alvarez et al. 2016).

### Ethanol production

Fermentation by cell cytoplasmic extracts (0.5 mg prot. mL^−1^) was measured in 0.1 M MES-TEA, pH 7.0, 1.8 mM NAD plus either glucose or glycerol and incubated at 30 °C for 0, 2.5, 5 or 10 min. The reaction was stopped with 30% TCA, 0.1 mL and neutralized with NaOH. Ethanol was measured adding a 10 µL aliquot (0.005 mg) of the supernatant to 0.2 mL 114 mM K_2_HPO_4_, pH 7.6. After 1 min, 30 μg ADH mL^−1^ was added, the sample was incubated for 30 min and O.D. was determined at 340 nm in a POLARstar Omega. Ethanol is reported as μmol ethanol (mg prot)^−1^ (Araiza-Olivera et al. 2013).

### ATP concentration

ATP was measured in cytoplasm extracts resuspended to 0.025 mg protein in 0.15 mL reaction buffer (20 mM KH_2_PO_4_, 40 mM Na_2_HPO_4_, 80 mM NaCl, 1 mM MgSO_4_). An ATP calibration curve was prepared freshly each day using lyophilized luciferase (Sigma-Aldrich). Luciferase was prepared following instructions by the provider and 0.02 mL was added to each sample in a 96-well microplate. Bioluminescence was detected in a POLARstar Omega luminometer (BGM LABTECH, Offenburg, Germany). [ATP] was reported as µmol (mg prot)^−1^ (Palikaras and Tavernarakis 2016; Mendoza-Hoffmann et al. 2018).

### Susceptibility to hydrogen peroxide-mediated damage

The effect of [H_2_O_2_] on the viability of *S. epidermidis* was determined as previously reported (Macvanin and Hughes 2010). Briefly, cells were adjusted to an O.D. = 0.1 (600 nm) and then H_2_O_2_ (0 to 25 mM as indicated) was added to the reaction mixture. After 30 min, serial dilution of the cultures was performed in 0.9% NaCl and 10 µL of the 1:1000 diluted sample was plated in LB, 2% glucose agar plates and incubated 24 h at 37 °C. Colony forming units (CFU) mL^−1^ were counted. The sample taken before H_2_O_2_ addition was assigned as 100%. The average of three experiments is shown with SD. ANOVA test and Tukey’s multiple comparison-test were used. Significance was *P < 0.0001.

### Biofilm formation and detection

Biofilm generation was measured in sterile Costar 96-well polystyrene plates as previously reported (Calà et al. 2015; Uribe-Alvarez et al. 2016). Briefly, in each well, 0.4% crystal violet in 33% glacial acetic acid was mixed with the indicated, inhibitors sodium cyanide (NaCN) (100 µM), butane-1,4-bisphosphate (B1,4BP) (1 mM) or, carbonyl cyanide *m*-chlorophenyl hydrazone (CCCP) (0.1, 0.5, or 1 µM, as indicated). Then bacteria were added to O.D. 0.02. Final volume 200 μL. The plate was incubated 24 h at 37 °C with 5% CO_2_. After incubation, wells were washed twice with 200 µL phosphate-buffered saline (PBS) to remove non-adherent bacteria. Plates were dried for 1 h at 60 °C, stained with 0.4% crystal violet for 10 min and washed under running tap water to remove excess stain. Absorbance (492 nm) was measured using a microplate reader (Polar Star Omega, BMG Labtech). Each sample was tested in three independent triplicate experiments and compared against the non-treated control using one-way variance analysis (ANOVA) plus Dunnett’s post hoc test.

## Results

Oxygen is among the most important factors driving evolution (Lane 2002). Its partial reduction products, the reactive oxygen species (ROS) destroy nucleic acids, proteins and membranes (Ezraty et al. 2017). Thus, to profit from its remarkable electron acceptor properties, organisms have to deal carefully with the dangerous oxygen molecule (Lane 2002; Rosas-Lemus et al. 2016b). *S. epidermidis* lives in hypoxic/anoxic environments, although it can adapt to high [O_2_]. In order to follow the metabolic adaptation of *S. epidermidis* it was cultivated at different [O_2_]. After 24 h under aerobic conditions biomass yield was 8.58 g/L, three times higher than under microaerobiosis, 2.11 g/L or anaerobiosis, 1.75 g/L.

**Fig. 1:**
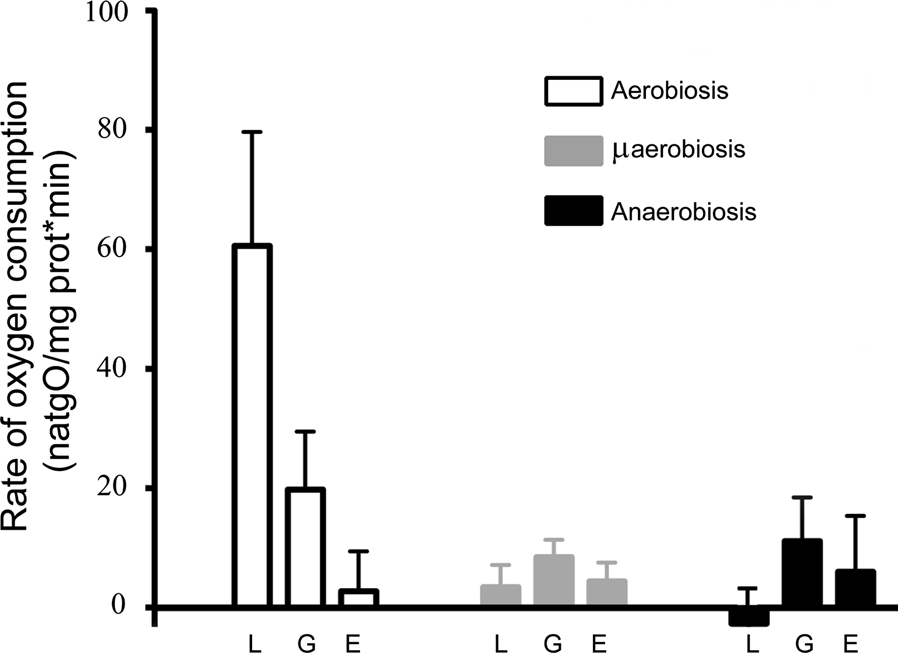
Rate of oxygen consumption by *S. epidermidis* in the presence of different respiratory substrates. Experimental conditions: 10 mM HEPES (pH 7.4). As indicated, substrates were: L: 10 mM lactate; G: 40 mM glucose or E: 33 mM ethanol. Cells were grown at different [O_2_] as follows: aerobic (empty bars), microaerobic (gray bars) and anaerobic (black bars)

In order to further explore the basis for biomass yield variations at different [O_2_], the activity of the respiratory chain from *S. epidermidis* grown at different [O_2_] was measured (Fig. 1). As expected from previous respiratory chain protein expression results (Uribe-Alvarez et al. 2016), the ability of cells to consume oxygen was proportional to [O_2_] in the growth medium. In aerobic conditions and in the presence of lactate the rate of oxygen consumption was 70 natgO (mg prot. min)^−1^, at least five times higher than in microaerobic media, where the rate was 5 natgO (mg prot. min)^−1^ or in those grown under anaerobic conditions, where it was negligible (Fig. 1). Under normoxia the best respiratory fuel was lactate, which was oxidized around three times as fast as glucose or ethanol (Fig. 1).

**Fig. 2:**
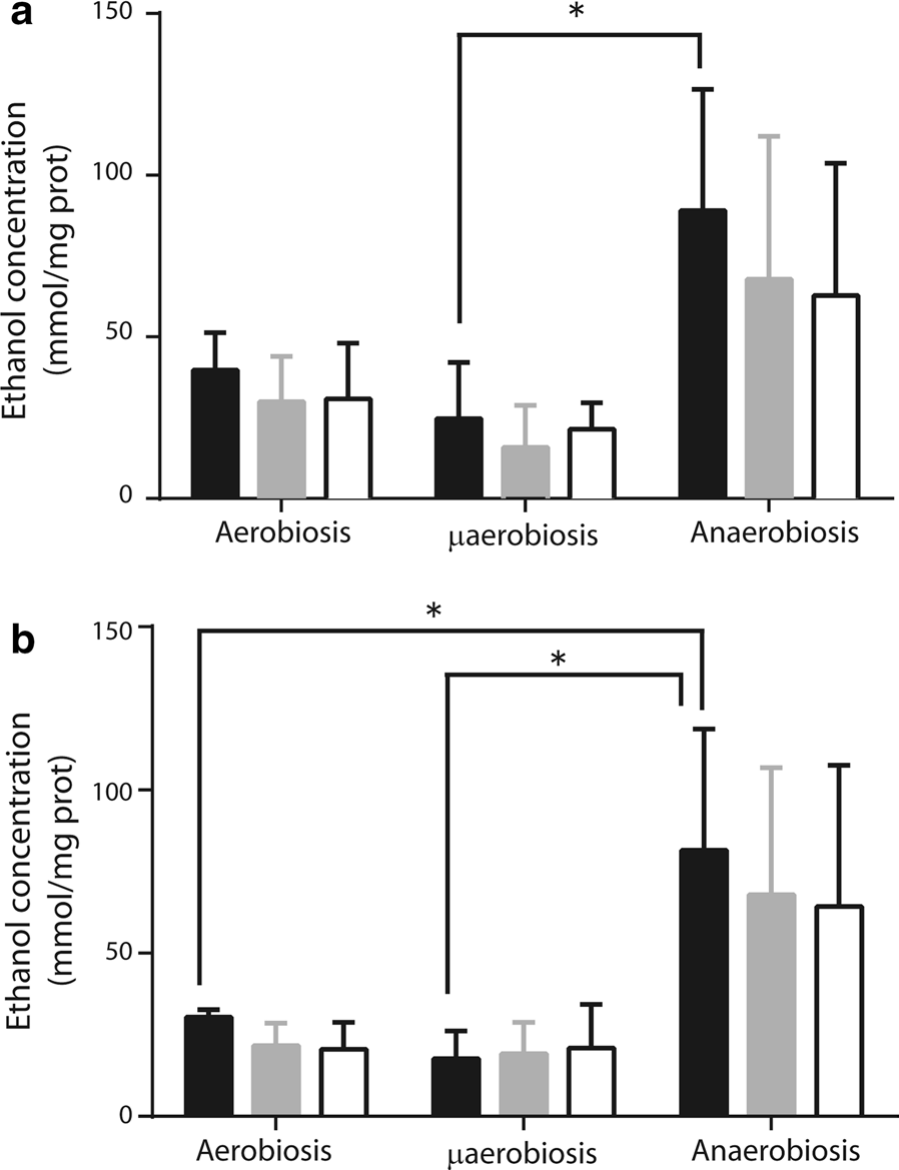
Fermentation by *S. epidermidis* grown at different [O_2_]. Cytoplasmic extracts were obtained from *S. epidermidis* grown under aerobic, microaerobic or anaerobic conditions. Fermentation by cell cytoplasmic extracts (0.5 mg prot. mL^−1^) was measured using **a** 20 mM glucose or **b** 20 mM glycerol. Samples were incubated at 30 °C for: 2.5 min (black columns), 5 min (gray columns) or 10 min (white columns). Results are reported as μmol ethanol per mg protein. Tukey’s comparison test was used to determine significant differences (*P < 0.05)

In *S. epidermidis* respiratory chain activities correlated with growth rates. However, it was reasoned that in hypoxia glycolysis may constitute an important source of energy (Somerville and Proctor 2009). Furthermore, as *S. epidermidis*, normally lives at low [O_2_], fermentation may be the preferred energy-yielding pathway in this bacterium. To test this, *S. epidermidis* was grown at different [O_2_] and ethanol production from either glucose (Fig. 2a) or glycerol (Fig. 2b) was measured at 2.5, 5 and 10 min of incubation. Both substrates were equally efficient. However, at different [O_2_] large variations in the rate of fermentation were observed: bacteria from anaerobic media were the most active, (Fig. 2), suggesting that fermentation increases as [O_2_] decreases.

**Fig. 3:**
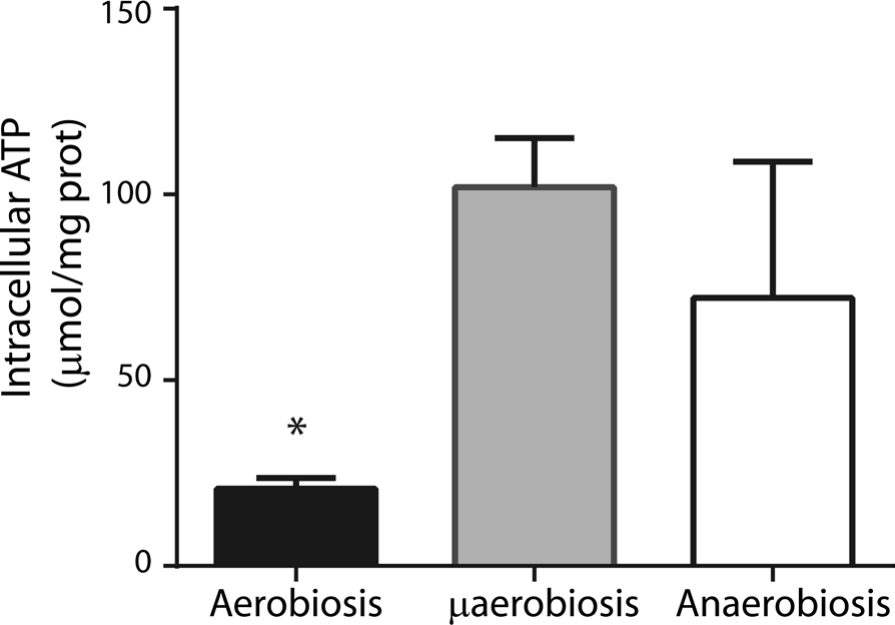
Intracellular ATP concentrations in *S. epidermidis* grown at different [O_2_]. Cells were grown at different [O_2_] in LB plus glucose. Cytoplasmic extracts were obtained from each of these cultures and used to measure intracellular ATP. ATP concentration was estimated using luciferase and interpolating into a standard curve (see “Materials and methods”). The average of three experiments is shown with SD. * indicates significant difference *P* < 0.05

In *S. epidermidis*, increasing [O_2_] increased the rate of oxygen consumption while fermentation was inhibited. To determine which of these pathways produced more energy, the concentration of ATP ([ATP]) was measured in *S. epidermidis* grown under normoxia, hypoxia or anoxia (Fig. 3). Contrary to what we expected from the low growth rate and the slow respiratory activity observed, in hypoxia- and anoxia-grown cells, [ATP] was higher than in normoxia as aerobiosis, [ATP] increased roughly five times in hypoxia and three times in anoxia as compared to normoxia (Fig. 3).

**Fig. 4:**
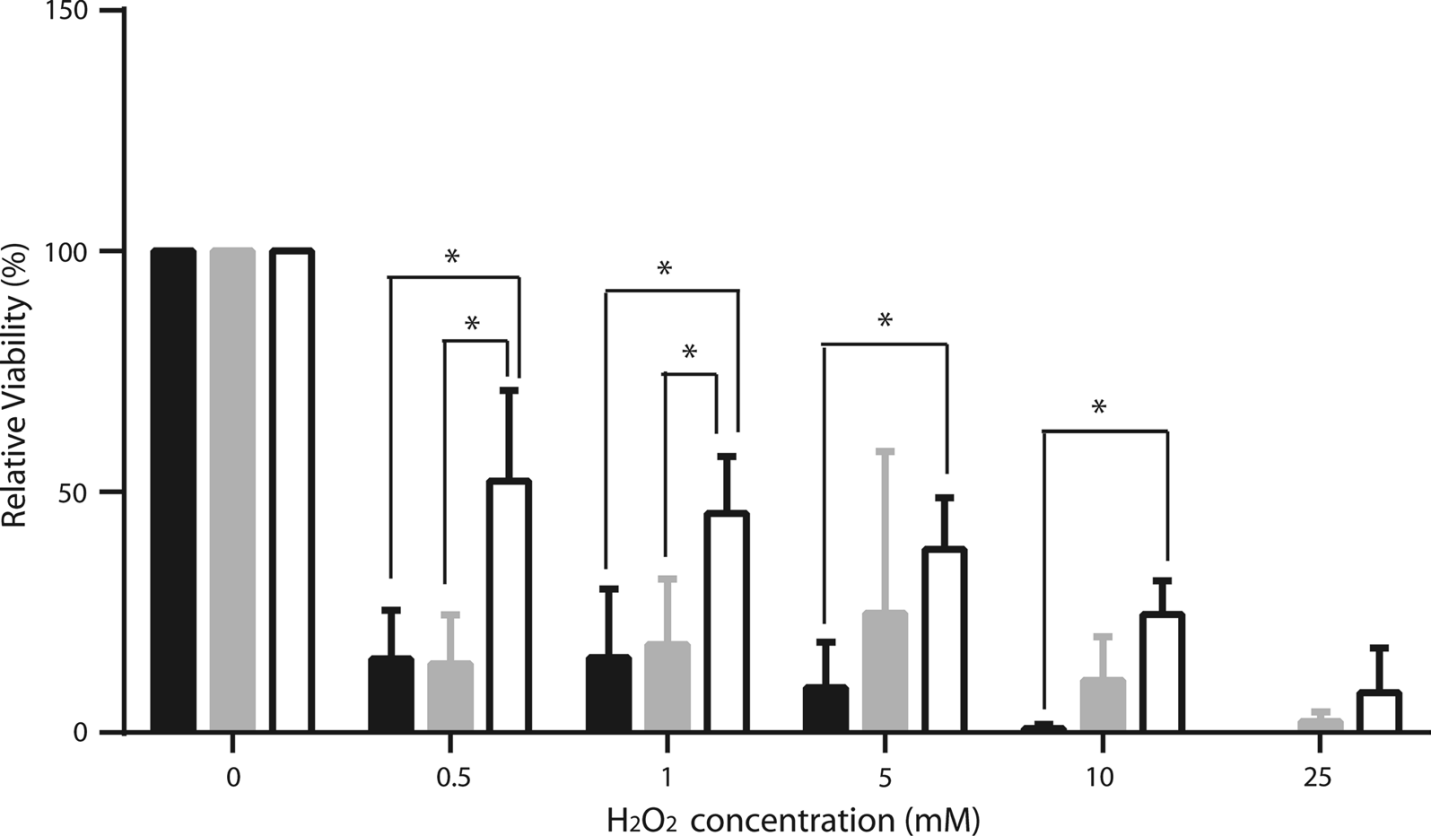
H_2_O_2_ effect on cellular viability. *S. epidermidis* susceptibility to hydrogen peroxide was determined using 0, 0.5, 1, 5, 10 or 25 mM H_2_O_2_ in each group: aerobiosis (black bar), microaerobiosis (gray bar) or anaerobiosis (white bar). After 30 min of incubation with H_2_O_2_, the samples were diluted 1:1000, 10 µL were taken and plated in LB plus 2% glucose-agar. CFU/mL were counted. Samples without treatment were assigned as 100% viable cells. The average of three experiments is shown with SD. Significance *P < 0.0001

In *S. aureus* a deficient respiratory chain confers resistance to H_2_O_2_ toxicity (Painter et al. 2017), suggesting that anaerobiosis-adapted cells resist oxidative stress better. Thus, we decided to test *S. epidermidis* grown at different [O_2_] for its sensitivity to H_2_O_2_ (Fig. 4) (Lobritz et al. 2015). Even at the lowest concentrations of H_2_O_2_ we used (0.5 mM), viability decreased in all cells. Aerobic-grown cells exhibited the poorest survival rates, while cells grown under anaerobiosis survived best, such that even at the highest H_2_O_2_ concentration tested (25 mM H_2_O_2_) a small amount of viable cells was detected (Fig. 4). The increase in sensitivity to ROS observed in aerobically grown *S. epidermidis* was probably due to increased expression of the redox enzymes in the respiratory chain (Uribe-Alvarez et al. 2016). These redox enzymes contain different coenzymes and prosthetic groups, which normally become free radicals during their catalytic cycle (Quinlan et al. 2013; Rosas-Lemus et al. 2016b). Thus, as reported for *S. aureus* (Painter et al. 2017) at high [O_2_] *S. epidermidis* expressed an active respiratory chain and its sensitivity to H_2_O_2_ increased.

**Fig. 5:**
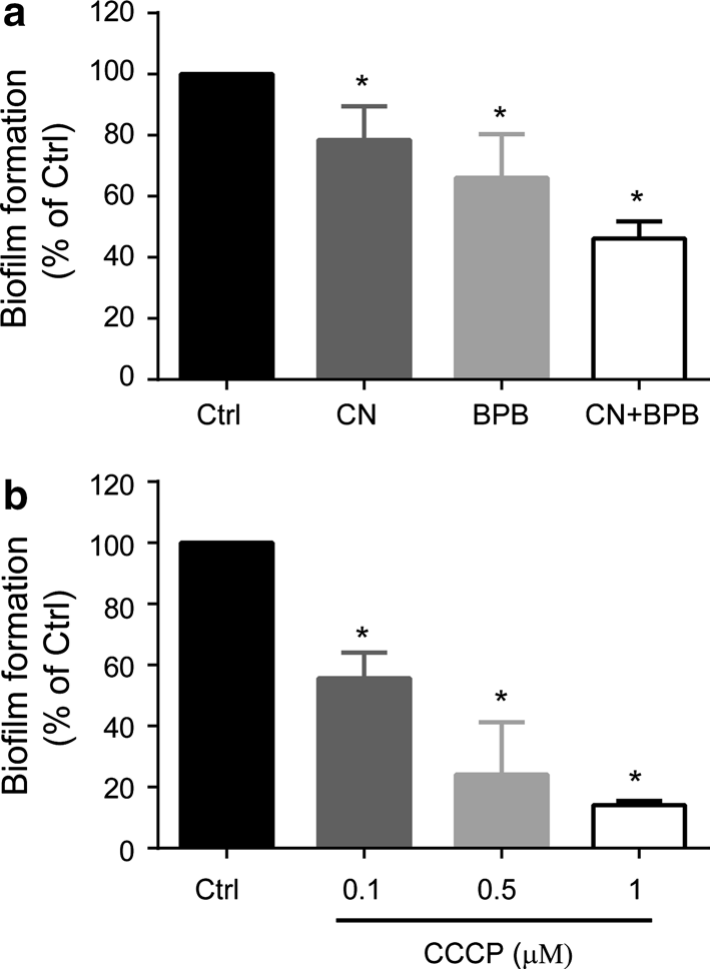
*In vitro* biofilm inhibition assay. *S. epidermidis* was grown under microaerobic conditions. **a** Different metabolic inhibitors were added as indicated: 100 µM NaCN, 1 mM B1,4BP or both inhibitors. **b** Different concentrations of the uncoupler CCCP (0.5, 1.0 and 1.5 µM) were added to deplete ATP. After 24 h of incubation biofilm generation was evaluated by measuring the absorbance at 492 nm with a microplate reader. Each sample was compared with the control (without additions). Statistics were applied using ANOVA and Dunnett’s post hoc test. Significance *P < 0.0001

The highest [ATP] was detected in cells grown at low [O_2_], which exhibited a slow growth rate. This seemingly contradictory situation may be explained by proposing that when *S. epidermidis* finds a low [O_2_], which resembles that found in its normal niche, it makes an effort to attach itself to a surface, redirecting its energy use from growth to produce polysaccharides and proteins for biofilm generation (Beenken et al. 2004; Lewis 2007). To analyze whether biofilm was dependent on [ATP], *S. epidermidis* was grown under hypoxia and in the presence and absence of different metabolic inhibitors. In hypoxic grown-cells both oxidative phosphorylation and fermentation are active. It was observed that cells incubated in the presence of the respiratory chain inhibitor cyanide or the glycolytic inhibitor 1,4-bisphosphobutane, formed smaller biofilms than the control and that addition of both inhibitors led to even less biofilms (Fig. 5a). This would suggest that biofilm formation activity is proportional to [ATP]. In addition, the uncoupler CCCP was used at concentrations below those where it killed cells (Result not-shown), observing that biofilm generation decreased further as uncoupler concentration increased (Fig. 5b). These results suggest that, regardless of its source, in *S. epidermidis* high [ATP] is needed to form biofilms.

## Discussion

Antibiotic-resistant strains of *S. epidermidis* are increasingly found in nosocomial infections (Chabi and Momtaz 2019). Implant removal due to *S. epidermidis* biofilm colonization is also quite frequent (Gristina 1987; Raad et al. 1998). *S. epidermidis* is frequently found in coagulase-negative staphylococci-caused prosthetic valve infective endocarditis cases (Mack et al. 2013), in 30–43% implant infections (Zimmerli et al. 2004) and in 50–70% catheter-related infections (von Eiff et al. 2002). Understanding the physiology of the bacterium is a must in order to design new treatment and prevention methods (Uribe-Alvarez et al. 2016). In biofilms, *S. epidermidis* cells are protected from the host. Thus, it is most important to analyse the association and specialization processes of the cells involved in the genesis of biofilms.

Diverse facultative bacteria adapt to wide [O_2_], differentially expressing redox enzymes in its respiratory chain. *S. epidermidis* does express different enzymes at varying [O_2_] (Uribe-Alvarez et al. 2016). Aerobic metabolism enabled cells to grow more (Baez and Shiloach 2014). Still, enhanced growth resulted in higher sensitivity to H_2_O_2_, suggesting that high contents of redox enzymes make cells vulnerable to ROS. Indeed, when grown at high [O_2_], sensitivity to ROS is enhanced in *S. aureus* and *Enterococcus faecalis*, while their mutant counterparts, lacking an efficient respiratory chain resist ROS better (Painter et al. 2017).

When exposing *S. epidermidis* grown in different [O_2_] to oxygen peroxide, we observed a similar phenomenon: cells grown in hypoxic or anoxic environments, which exhibited low respiratory rates were more resistant to oxygen peroxide (Fig. 4). Thus, as in *S. aureus*, the lack of an efficient respiratory chain in *S. epidermidis* enabled cells to survive ROS. This is probably useful when bacteria detached from a biofilm reach other tissues where they may be confronted with the oxidative burst generated by the immune system (Jensen et al. 1992).

The rate of oxygen consumption in aerobic grown cells was highest when lactate was the substrate. This is probably due to the direct donation of electrons to the menaquinone pool by lactate dehydrogenase (Götz and Mayer 2013; Kane et al. 2016). The slower rates observed for alcohol, may be due to an additional step as alcohol dehydrogenase electrons are first donated to Ndi2 (Artzatbanov and Petrov 1990). The rate of respiration was also slow for glucose, probably for the same reason, as intermediaries have to undergo many reactions before releasing electrons to the respiratory chain (Ferreira et al. 2013). In contrast, under anaerobiosis, lactate-dependent oxygen consumption disappeared completely while a small rate of glucose-dependent oxygen consumption was still present. In contrast, in *S. aureus* increased lactate dehydrogenase expression anaerobiosis has been reported (Fuchs et al. 2007).

The normal habitat for *S. epidermidis* is the microaerobic environment found in different epidermic and dermic layers (Peyssonnaux et al. 2008). One strategy *S. epidermidis* uses when confronted with high [O_2_] is the differential expression of a diverse number of redox enzymes in the respiratory chain. Reports indicate that when microaerophilic or anaerophilic bacteria find a suitable environment, they react manufacturing proteins and polysaccharides that enable them to form biofilms and attach to surfaces at low [O_2_]. Avoiding high [O_2_] involves both, anchoring in low oxygen environments and building biofilms as barriers against penetration of ROS or toxic substances (Palikaras and Tavernarakis 2016). Metabolic adaptation has also been reported for *Neisseria gonorrhoeae*, when it is stimulated to form biofilms. A proteomic analysis of *N. gonorrhoeae* biofilms evidenced up-regulation of proteins involved in anaerobic metabolism such as glycolysis and TCA cycle plus increased expression of those proteins involved in biofilm generation like pilus-associated proteins (Phillips et al. 2012). In addition, some oxidative stress genes are required for normal biofilm formation in *N. gonorrhoeae* (Falsetta et al. 2011).

The increase in ATP prior to biofilm formation has been reported in others bacterium. *Bacillus brevis and Escherichia coli* react to substrate depletion by adhering to glass surfaces and at the same time increase [ATP] two to fivefold as compared to planktonic cells (Hong and Brown 2009). So, the conditions where bacteria need to make biofilms promote saving ATP even at the expense of the growth rate. ATP is most likely needed to synthesize the extracellular proteins and the polysaccharide fibers that anchor cells to surfaces and to each other. Inhibiting ATP production in micro- or anaerobic conditions by adding cyanide or 1,4-bisphosphobutane resulted in a reduced biofilm formation (Fig. 5). This phenomenon is also observed when treating *S. epidermidis* with the nitrate reductase inhibitor methylamine in anaerobic conditions (Uribe-Alvarez et al. 2016). In contrast, in aerobiosis cyanide promotes biofilm formation (Uribe-Alvarez et al. 2016).

**Fig. 6:**
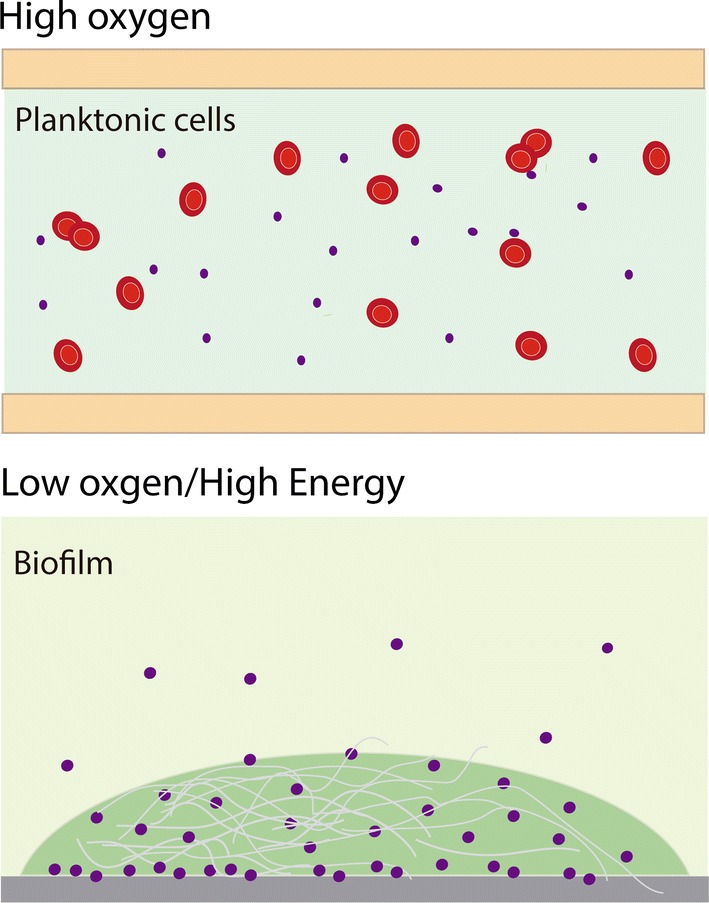
Cartoon depicting the shift that *Staphylococcus epidermidis* makes when [O_2_] decreases in the growth medium. When high oxygen concentrations are found in the medium, *S. epidermidis* are planktonic cells and flow with the blood (top). In contrast, under micro- or anaerobic conditions cells shift to a fermentative metabolism and accumulate ATP adhering to a suitable surface (e.g. epithelia, catheters, artificial valves) and eventually forming a biofilm. In this state the cells exhibit more resistance to H_2_O_2_ mediated damage. Excess ATP is probably used to produce adhesion proteins and poly-*N*-acetylglucosamine (gray fibers in the illustration) (bottom)

Even when facultative bacteria such as *S. epidermidis* survive at high [O_2_], their habitat in the skin is hypoxic to anoxic. While they survive in aerobic environments their susceptibility to ROS-mediated damage and possibly to attack by macrophages increases. They thus present an oxygen avoidance behavior, anchoring and associating in hypoxic environments (Fig. 6). Learning how avoidance works in *S. epidermidis* and other bacteria would impact both the physiologic and therapeutic field.

Aiming to understand such rise in ATP, we found that other bacteria, e.g. *Bacillus brevis* and *Escherichia coli*, react to substrate depletion by adhering to glass surfaces and at the same time increase [ATP] two- to fivefold in comparison to planktonic cells (Hong and Brown 2009). In this regard, it has been reported that hypoxic stimuli induce biofilm formation in *S. epidermidis* (Uribe-Alvarez et al. 2016).
